# Comparison of the effects of retroperitoneoscopic ureterolithotomy and ureteroscopic lithotripsy in the treatment of upper ureteral calculi

**DOI:** 10.1097/MD.0000000000027328

**Published:** 2021-09-24

**Authors:** Sheng-Lin Gao, Hao Wu, Quan-Xin Su, Zi-Yi Zhang, Ze Zhang, Chao Lu, Li-Feng Zhang, Li Zuo

**Affiliations:** a Department of Urology, The Affiliated Changzhou No. 2 People's Hospital of Nanjing Medical University, Changzhou, China; b Dalian Medical University, Dalian, China.

**Keywords:** retroperitoneoscopic ureterolithotomy, upper ureteral calculi, ureteral calculus, ureteroscopic lithotripsy

## Abstract

This study compares the efficacy of retroperitoneoscopic ureterolithotomy (RPUL) and ureteroscopic lithotripsy (URL) in the treatment of upper ureteral calculi.

The clinical data of 150 patients with upper ureteral calculi who underwent RPUL and 136 patients who underwent URL between January 2014 and October 2019 were retrospectively analyzed. The operation time, postoperative hospital stay, operation success rate, stone clearance rate, and surgical complications were evaluated between the two groups.

For the RPUL and URL groups, respectively, the average operation time was 74.5 ± 24.6 minutes and 54.5 ± 13.2 minutes; the postoperative hospital stay was 5.8 ± 1.4 days and 3.2 ± 1.2 days; the operation success rate was 96.0% (144/150) and 85.3% (116/136); the incidence rate of complications was 3.5% (5/144) and 17.5% (18/103); and the stone clearance rate was 100% (144/144) and 88.8% (103/116), which were all statistically significant (*P* < .05).

Both RPUL and URL had the advantages of low trauma and fast recovery rate for patients with upper ureteral calculi. However, patients who underwent RPUL showed higher success and fewer complication rate. RPUL might be a safe and effective laparoscopic method for the treatment of patients with upper ureteral calculi.

## 1 Introduction

Ureteral calculi are a common concern in urology. The increasing prevalence of urolithiasis is linked to the increasing incidence of obesity, diabetes, and metabolic syndrome.^[1]^ In response, the treatment of ureteral stones is also developing rapidly.^[2]^ At present, there is a variety of minimally invasive treatment methods for ureteral calculi, including ureteroscopic lithotripsy (URL), which has gradually replaced traditional open surgery.^[3,4]^ However, using this treatment method still gives rise to complications, and has a definite residual rate of calculi in the upper segment of the urinary catheter.^[5]^ With the widespread development of laparoscopic surgery in the field of urology, retroperitoneoscopic ureterolithotomy (RPUL) has emerged as a new treatment method for ureteral stones.^[6]^ From January 2014 to October 2019, we performed a total of 150 RPUL procedures and 136 ureteroscopic pneumatic lithotripsy and holmium laser lithotripsy procedures to treat upper ureteral calculi. The therapeutic effects of these procedures were compared.

## 2 Material and methods

### 2.1 Clinical data

A total of 286 cases were evaluated, consisting of 150 cases of RPUL and 136 cases of upper ureteral calculi treated with ureteroscopic pneumatic ballistic lithotripsy and holmium laser lithotripsy. Basic data for the 2 groups were shown in Table 1. Except for sex, the 2 groups of patients showed no statistically significant differences in the age, body mass index, stone size, and degree of hydronephrosis (*P* > .05), making them valid for comparison. The criteria for case selection were^[7]^: ① patients who failed the extracorporeal shock wave lithotripsy (ESWL) treatment; ② stone diameter of >1.5 cm; and ③ stone present in the ureter for more than 8 weeks or with inflammatory polyps. All patients underwent routine B-ultrasound and plain abdominal radiography. Confirmatory computed tomography examination was performed for those with poor imaging findings. In order to prevent deep venous thrombosis of lower extremities, patients in both groups were given subcutaneous injection of “natriuretic heparin calcium” 24 hours after operation, once a day.

**Table 1:** Comparison of basic information between RPUL group and URL group.

		Sex					
	No. of cases	Male (n)	Female (n)	Age (years)	BMI (kg/m^2^)	Stone size (cm)	Ipsilateral hydronephrosis (cm)
RPUL group	150	90	60	54.80 ± 12.26	23.80 ± 2.70	1.56 ± 0.54	2.78 ± 0.76
URL group	136	63	73	54.25 ± 13.55	22.92 ± 2.72	1.48 ± 0.44	2.68 ± 0.72
*P* value		.021		.417	.195	.172	.424

BMI = body mass index, RPUL = retroperitoneoscopic ureterolithotomy, URL = ureteroscopic lithotripsy.

### 2.2 Surgical methods

Patients in the RPUL group were placed under general anesthesia, and were positioned in a contralateral lying position. The skin was incised 2.0 cm on the iliac crest along the midaxillary line. Afterwards, the muscle layer was separated, and a homemade balloon was placed and then inflated to 600 mL volume capacity to expand the peritoneal space. The 1.0 cm and 0.5 cm cannula were placed under the costal margin of the posterior axillary line and the anterior axillary line, respectively, and the pressure of carbon dioxide pneumoperitoneum was maintained at about 15 mm Hg (1 mm Hg = 0.133 kPa). An electric hook or harmonic scalpel was placed inward along the psoas muscle to find the ureter segment with the stone, while the ureter segment above the stone was secured with grasping forceps. If the stone was close to the renal pelvis, the perirenal fascia of the lower pole of the kidney was opened, and the ureteral stone was sought medial to the lower pole of the kidney. The ureter segment at the stone site was incised, and the stone was removed. For cases with associated ureteral polyps, the polyps were also removed. A double-J tube was then placed through the ureteral incision. Finally, the ureteral incision was sutured with 1-2 stitches using a 4-0 delayed absorbable suture. The suture we used was braided absorbable suture UL-203 from COVIDIEN Company.

Patients in the URL group underwent epidural anesthesia and were placed in the lithotomy position. A semi-rigid F 8.0 to 9.8 Wolf ureteroscope was inserted from the urethral meatus to the bladder, thereafter a guide wire or ureteral catheter was inserted into the ureteral orifice. Afterwards, the ureteroscope was inserted along the guide wire while flushing. After the stones were spotted, a pneumatic lithotripsy rod was inserted to break the stones with continuous pulses, or a medical holmium laser fiber was inserted with a holmium laser energy of 0.8 to 1.0 J and a frequency of 10 to 20 Hz. The stones were broken, and the stones with a diameter of >0.3 cm were removed with a stone forceps, while the small ones were flushed out with water flow, after which a 6 F ureteral double-J stent was placed.

All patients of both groups were treated with double-J stent during operation. We routinely informed patients to come back to the hospital for re-examination 6 to 12 months after the stent was removed. We followed up these 2 groups of patients 6 to 36 months after operation. For some patients who do not come to the hospital at the prescribed time, we followed up over the telephone.

### 2.3 Observation indicators

The operation time, length of postoperative hospital stay, surgical success rate, stone clearance rate, and surgical complications were all observed and analyzed.

### 2.4 Statistical methods

Statistical analyses were performed using the SPSS 22.0 statistical software (IBM Corporation, USA). All data were expressed in mean ± standard deviation and a *t* test was performed. Χ^2^ test was used for counting data. The differences in the parameters were tested at a significance level of 0.05.

## 3 Results

In the RPUL group of 150 patients, 144 patients underwent the surgery successfully, upper ureteral stones slipping into the renal pelvis in 3 patients, and 3 patients were changed to open surgery for unclear intra-operative bleeding fields. Perioperative complications in the RPUL group were: 5 cases of urine leakage. These patients were healed spontaneously after 7 to 11 days. A total of 95 patients were followed up for 6 to 36 months. The stone clearance rate was 100%. Six patients with stone recurrence were treated with ESWL. During the process of follow-up, 3 patients developed ureteral stricture (Table 2). Among them, 2 patients were treated with long-term indwelling stent. One patient chose ureteral stricture resection and re-anastomosis.

**Table 2:** Comparison of efficacy between RPUL group and URL group.

	No. of cases	The operation time (min)	The postoperative hospital stay	The operation successful rate (%)	The stone clearance rate (%)	The incidence rate of complications (%)	The incidence rate of ureteral stricture (%)
RPUL group	150	74.5 ± 24.6	5.8 ± 1.4	96.0 (144/150)	100.0 (144/144)	3.5 (5/144)	3.2 (3/95)
URL gorup	136	54.5 ± 13.2	3.2 ± 1.2	85.3 (116/136)	88.8 (103/116)	17.5 (18/103)	2.2 (2/90)
*P* value		<.001	<.001	.002	<.001	<.001	.951

RPUL = retroperitoneoscopic ureterolithotomy, URL = ureteroscopic lithotripsy.

There were 136 cases in URL group, including 53 cases of pneumatic lithotripsy and 83 cases of holmium laser lithotripsy. One hundred sixteen patients underwent the surgery successfully. Eleven patients were changed to laparoscopic surgery (6 case in 11) or open surgery (5 case in 11) for the failure of ureteroscopy caused by ureteral distortion, stricture, or polyp wrapping below the stone. Stones entering the renal pelvis in 9 patients. Among them, 3 cases were converted to open surgery and 6 cases were treated with ESWL. There were significant differences in the operation time, postoperative hospital stay, success rate, stone clearance rate, and the incidence rate of complications between RPUL and URL group (*P* < .05, Table 2). After the primary URL, there were 13 cases of residual stones in the URL group. These patients were treated with flexible URL or ESWL. Complications in the URL group included ureteral orifice injury in 3 cases, ureteral submucosal false passage in 5 cases, and ureteral perforation in 5 cases. These patients were treated with double-J tube drainage. Moreover, there were acute pyelonephritis in 3 cases, who were cured after anti-infective treatment. Two cases developed aggravated hydronephrosis and they were treated with open surgery. During the process of follow-up, 90 patients in URL group were followed up for 6 to 36 months, wherein 87 cases of hydronephrosis were improved, and 7 cases of stone recurrence were treated with ESWL. Two patients developed ureteral stricture (Table 2) and were treated with open surgery.

## 4 Discussion

At present, methods for treating upper ureteral calculi include ESWL, URL, nephroscopic lithotripsy, laparoscopic surgery, and open surgery. Satisfactory results can be obtained using ESWL in most patients, but its efficacy can be influenced by many factors, such as the composition, location, and size of the stones.^[8]^ Patients with poor ESWL treatment usually require open surgery in the past, resulting in long incisions, large trauma, and slow postoperative recovery. With the development of minimally invasive surgery, laparoscopic and ureteroscopic surgery gradually replaced open surgery as an important means to treat upper ureteral stones.

RPUL was first successfully performed by Raboy et al^[9]^ in 1992. RPUL has become another option for minimally invasive treatment of ureteral stones due to many advantages including lower trauma and faster postoperative recovery. Moreover, retrograde intra-renal holmium-laser was revealed to be an effective and safe treatment for urinary system disease including symptomatic renal sinus cysts.^[10,11]^ We observed that identifying and confirmation of the stone was the key to a successful operation. Psoas muscle and lower pole of the kidney were important anatomical landmarks for finding the ureter. The ureter segment with stone was significantly bulging, with surrounding adhesions, and a hard texture during clamping, while the ureter above the stone was dilated. For stones near the renal pelvis, the perirenal fascia could be incised dorsally in the lower pole of the kidney to separate the dilated renal pelvis and ureter. Ureterotomy could be performed with an electric coagulation hook or a self-made endoscopic knife. The ureter segment above the stone was clamped with grasping forceps to fix it in place. The full thickness of the ureter from above the stone into the stone area was incised longitudinally. The incision length was slightly longer than the length of the stone. The stone was fully freed with dissecting forceps and then removed. We also found that the placement of a ureteral double-J tube can not only maintain the patency of the ureter but can also reduce the probability of ureteral stricture after operation. According to the method of Xu et al,^[12]^ a 4F ureteral catheter could be inserted into a 7F double-J tube as the internal stent, and a double-J tube was placed from the ureteral incision. Methylene blue normal saline could be filled in the bladder to determine whether the distal of stent had entered the bladder. Since laparoscopy provides a 2-dimensional field of vision and with visual deviation, it was relatively difficult to suture and tie knots under this process. It is necessary for urologists to master the operating skills of abdominal cavity. We used a 4-0 absorbable suture, and the length of suture should not exceed 10 cm. In the process of stitching, it is necessary to pay attention to the layer and edge distance of the suture, leaving a tail of about 2 cm to tie a knot. The needle holder should wrap the suture clockwise for 2 turns behind the suture and then the tail was clamped to pull out. Then, it was significant to wrap the suture clockwise for 1 turn to tighten the tail.

The advantages of ureteroscopy in the treatment of ureteral calculi include the use of natural orifices, prevention of wounds, and management of ureteral calculi simultaneously. Ureteroscopy is thought to be effective in the treatment of middle and lower ureteral stones, however, the success rate in treating upper ureteral stones was significantly lower than that of middle and lower ureteral stones.^[13,14]^ If ureteroscopy was used to treat upper ureteral calculi, the distance into the lens was longer. The ureteroscope must pass through 2 narrow sections. It was very difficult for the ureteroscope to locate the stone if the ureter was narrow or twisted, especially for male patients with prostate hyperplasia. In addition, upper ureteral stones were closer to the renal pelvis, and the stones can easily move into the renal pelvis by water pressure.^[15,16]^ Therefore, it was particularly significant for a urologist to master the skills and be experienced in the application of the ureteroscope. Out of the 136 cases of upper ureteral calculi that underwent ureteroscopy, 116 cases were successful, while 20 cases failed. Among them, 11 cases presented with difficulty in inserting the ureteroscope because the ureter underneath the calculi was twisted, narrowed, or wrapped by polyps. Another 9 cases were failed because the stones entered the renal pelvis and calyx. We revealed that successful insertion of ureteroscope was a pre-requisite for ureteroscopic treatment of upper ureteral calculi. It was also a key point to keep the stone fixed and prevent it from entering the renal pelvis. The ureteral cavity should be clearly evaluated when placing the ureteroscope. If the ureteral cavity cannot be seen clearly, it was necessary to adjust the angle and withdraw the ureteroscope properly. Blind entry and withdrawal of ureteroscope may lead to serious complications such as perforation and rupture of the ureter. When the stone was located, it was recommended to use the high head and low foot position. During the process of lithotripsy, low pressure perfusion and low-energy was also recommended, especially for holmium laser lithotripsy. It was a good choice to aim at the stone and crush the stone from the edge.

In the present study, there were no significant differences in the parameters, such as the age, body mass index, stone size, and degree of hydronephrosis between the 2 groups, except for sex. The rate of operation success between RPUL group and URL group was 96.0% versus 85.3% (*P* < .05). The operation time and postoperative hospital stay in the URL group were shorter than those in the RPUL group (*P* < .05). No patient with residual stones was observed in the RPUL group. However, 13 cases with residual stones were found in the URL group. They were advised to be treated with ESWL or flexible URL subsequently. The main complication in the RPUL group was urine leakage, which was considered to be caused by failure of ureteral suture in the early stage of surgery. Kumar et al^[17]^ previously compared RPUL and URL for the treatment of upper ureteral stones larger than 2 cm and observed that RPUL had a higher stone elimination rate and a lower complication rate compared with URL. These conclusions coincide with the findings of this study. With the maturity of laparoscopic techniques, the occurrence of urine leakage can be avoided by refined intra-operative ureteral sutures and the application of double-J tubes. In URL group, ureteral injuries were the main complication. The main reason was that the ureter was inserted blindly and forcedly during the process of ureteroscopy. The longer the stone incarceration, the more serious the local inflammatory reaction of the ureter would be. It was easy to cause ureteral perforation during the process of lithotripsy when handling inflammatory polyps.^[16]^ In URL group, ureteral injury was occurred in 13 patients. They were treated with double-J tube drainage without conversion to open surgery. Ureteral stricture was a long-term complication. In URL group, ureteral stricture was occurred in 2 patients. The possible reason may be related to ureteral polyps and ureteral perforation.

It is particularly important to choose different minimally invasive lithotripsy for various patients with upper ureteral calculi. URL is suitable for patients with calculi diameters less than 1.5 cm, especially for those without obvious stenosis or curvature at the distal end of the calculi. URL is also recommended for women or patients with bilateral ureteral calculi. RPUL is recommended for patients with calculi diameters more than 1.5 cm, especially for those with the past history of failed ESWL or ureteroscopy. URL is also suitable for patients with long stone incarceration, high stone density, and distal stone stenosis.

In conclusion, both RPUL and URL are effective methods for the treatment of patients with upper ureteral calculi. RPUL has high surgical success rate and stone clearance rate, with few surgical complications. URL has no need for surgical incision and fast postoperative recovery. RPUL might be a safe and effective laparoscopic method for the treatment of patients with upper ureteral calculi.

## Author contributions

LZ and LFZ conceived and designed this study, SLG and ZYZ wrote the main manuscript text, HW and QXS prepared tables, CL and ZZ acquired the case, SLG and LFZ revisited the manuscript. All the authors approved the submission of this study.

**Conceptualization:** Lifeng Zhang.

**Methodology:** Quan-Xin Su.

**Project administration:** Lifeng Zhang, Li Zuo.

**Resources:** Ze Zhang, Chao Lu.

**Software:** Hao Wu, Quan-Xin Su.

**Supervision:** Li Zuo.

**Writing – original draft:** Sheng-Lin Gao.

**Writing – review & editing:** Zi-Yi Zhang.
